# Integrated community-directed intervention for schistosomiasis and soil transmitted helminths in western Kenya – a pilot study

**DOI:** 10.1186/1756-3305-5-182

**Published:** 2012-08-31

**Authors:** Pauline NM Mwinzi, Susan P Montgomery, Chrispin O Owaga, Mariam Mwanje, Erick M Muok, John G Ayisi, Kayla F Laserson, Erick M Muchiri, W Evan Secor, Diana MS Karanja

**Affiliations:** 1 Neglected Tropical Diseases Branch, Center for Global Health Research, Kenya Medical Research Institute, 1578–40100, Kisumu, Kenya; 2 Ministry of Public Health and Sanitation, Division of Vector Borne and Neglected Tropical Diseases, Nairobi, Kenya; 3 Centers for Disease Control and Prevention, Division of Parasitic Diseases and Malaria, Atlanta, GA, USA; 4 KEMRI/CDC Research and Public Health Collaboration, Center for Global Health Research, Kenya Medical Research Institute (KEMRI/CDC), 1578–40100, Kisumu, Kenya

## Abstract

**Background:**

Schistosome and soil-transmitted helminth (STH) infections are recognized as major global public health problems, causing severe and subtle morbidity, including significant educational and nutritional effects in children. Although effective and safe drugs are available, ensuring access to these drugs by all those at risk of schistosomiasis and STHs is still a challenge. Community-directed intervention (CDI) has been used successfully for mass distribution of drugs for other diseases such as onchocerciasis and lymphatic filariasis. A national control programme is yet to be instituted in Kenya and evidence for cost-effective strategies for reaching most affected communities is needed. This study evaluated the effectiveness and feasibility of the CDI strategy in the control of schistosomiasis and STHs, in East Uyoma location, Rarieda district, a community of western Kenya that is highly endemic for both infections.

**Results:**

Pre-treatment prevalence of *S. mansoni* averaged 17.4% (range 5-43%) in the entire location. Treatment coverage in different villages ranged from 54.19 to 96.6% by community drug distributor (CDD) records. Assessment from a household survey showed coverage of 52.3 -91.9% while the proportion of homesteads (home compounds) covered ranged from 54.9-98.5%. Six months after one round of drug distribution, the prevalence levels of *S. mansoni,* hookworm and *Trichuris trichura* infections were reduced by 33.2%, 69.4% and 42.6% respectively.

**Conclusions:**

This study shows that CDI is an accepted and effective strategy in the mass treatment of schistosomiasis and STH infections in resource constrained communities in Kenya and may be useful in similar communities elsewhere. A controlled trial comparing CDI and school based mass drug administration to demonstarte their relative advantages is ongoing.

## Background

Schistosome and soil-transmitted helminth (STH) infections cause a huge burden of disease in the developing world [1], and have been associated with significant educational and nutritional effects [2,3]. School age children suffer from subtle morbidity such as anemia, chronic pain, diarrhea, exercise intolerance, growth stunting, undernutrition and impaired cognitive development, leading to poor school performance [3]. In children and adults, schistosomiasis mansoni can have nonspecific clinical manifestations such as bloody diarrhea and abdominal discomfort, and if untreated can lead to serious liver complications. While this burden is difficult to measure, deaths due to schistosomiasis have been estimated as high as 200,000 per year [3].

In Kenya, over 6 million people are estimated to be infected [1] and many more are at risk. The highest infection rates are found in adolescents aged 10–19 years, but adult workers in rural areas who are employed in activities associated with water contact are also affected [4-6]. Overall, the prevalence of schistosomiasis ranges from 5% to over 65% in communities in Kenya and contributes to significant morbidity [5-8]. In the Nyanza region, schistosomiasis is largely associated with Lake Victoria [9]. There are still many areas where the true burden with schistosomiasis is not well known as disease mapping has not been adequate. Recent efforts to map schistosomiasis in this setting [10-12] should continue to be encouraged. Prevalence levels among school children along the Lake shores ranged between 29-94% [10]. In another study, more than 50% of the children in schools located less than 1 kilometer from the lake were positive for *S. mansoni* infection. This prevalence decreased with increasing distance from the lake [9].

Often, schistosome infections co-occur with STHs. In Kenya, the prevalence of STHs is prominently attributed to *Ascaris lumbricoides*, hookworm and *Trichuris trichiura*[13,14]. It is estimated that approximately 10 million Kenyans are infected with STHs and over 12 million people living in rural endemic areas in the country are at risk of infection with these parasites. In Nyanza, up to 50% of school going children were infected with hookworm, ascarids, or trichurids [14] but there was no effect of lake proximity in the overall distribution of these STHs [9,14].

Despite the importance of schistosomiasis and STHs in Kenya, a National Control Program for the community has not been instituted. However, a national deworming program targeting school aged children was launched by both Ministers of Basic Education and Public Health and Sanitation, and implemented in 45 districts (total districts 254) in year 2009. Currently, school-based deworming is the primary approach being used for mass treatment of schistosomiasis and STHs and has been supported in a few areas in the country, mostly by local and international non-governmental organizations (NGOs). However, this approach is limited by low school enrolment in Kenya despite the introduction of free universal primary education in 2002 as poverty-related indirect costs prevent attendance by many children. Effective control by the school-based strategy may not be possible if other infected members of the community are not treated, such as fishermen, farmers in irrigation schemes, sand harvesters and mothers of child bearing age who can have high levels of infection but are not reached by school-based treatment programs. Involvement of communities, both for implementing school-based treatment and for increasing coverage to all those at risk, has not been effectively employed.

To improve access to treatment at affordable and sustainable costs and, where possible, to improve existing programs, community-directed intervention (CDI) has been presented as an alternative strategy for control of schistosomiasis and STHs. The CDI approach has been tried in several settings [15-18] and has worked successfully in many countries for the African Programme for Onchocerciasis Control (APOC) [19-21] as well as for lymphatic filariasis control along the Kenyan coast [22,23].

Effective and safe drugs for treatment of STHs are available in Kenya. However, drugs for treatment of schistosomiasis are not available in the health facilities in the country, and STH treatment is intermittently available in health facilities. Most individuals at high risk of morbidity do not have access to treatment because of the paucity of health infrastructure, lack of awareness about the disease, or cost. Therefore it is pertinent to evaluate if CDI would increase access to treatment among community members as well as increase coverage in school age children. We used the CDI approach for treatment against schistosomiasis and STHs in East Uyoma location of Western Kenya, an area along the shores of Lake Victoria highly endemic for schistosomais and STHs, to test CDI acceptability and effectiveness in reducing disease prevalence and burden.

## Methods

### Study area

**Figure 1:**
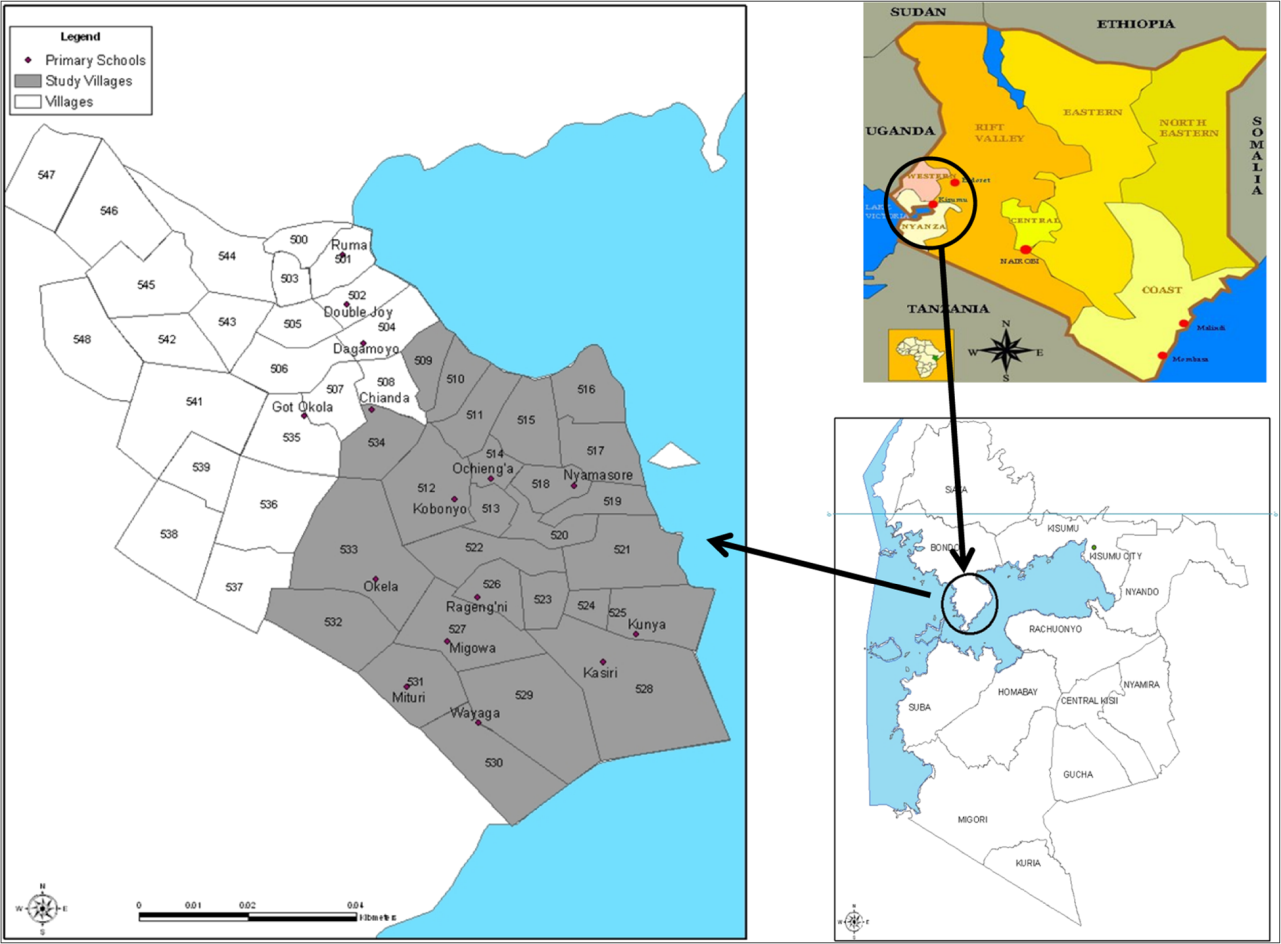
Map of East Uyoma in western Kenya showing study villages along the shores of Lake Victoria.

This study was conducted in a rural part of Nyanza Province, Western Kenya in Madiany Division, East Uyoma location, within the Health and Demographic Surveillance System (HDSS) platform. This HDSS was established in September 2001 by the Kenya Medical Research Institute and the CDC (KEMRI/CDC) [24]; (Figure 1). East Uyoma location has 25 villages. This study area borders Lake Victoria where earlier surveys had identified schistosomiasis prevalence levels between 30% to 80% among highly exposed individuals [5,6,9]. 

### Study design

### Mapping and census of study area

Village representatives (VR) who had participated in previous studies in the area were asked to help with a map updating exercise. This included confirming compound IDs and mapping any new compounds that had been established since the previous mapping by the KEMRI/CDC HDSS program. The coastline, which changes over time due to the receding waters of Lake Victoria, was also updated. A database identifying compounds, compound heads, and households in each compound was created and updated maps of the East Uyoma were generated, showing locations of schools and clinics, village boundaries, and other relevant features like rivers or creeks (Figure 1). This database was used to determine geographical treatment coverage and for mapping disease distribution. In total, there are 25 villages in the study area with 2046 compounds and an average 4.05 households per compound. After mapping the village, trained CDDs conducted the village census to determine the number of praziquantel and albendazole tablets required in his/her allocated area. Each CDD was assigned an average of 60 compounds. Compound heads were consented for inclusion of their home compounds in the census in preparation for drug distribution. The census was conducted by CDDs using pre-designed census booklets

### Prevalence survey

Parasitological surveys were conducted by the research team. Pre-treatment prevalence levels of *S. mansoni* infections, STH infections and anemia were assessed among standard 4 children aged 9–10 years old in all 10 schools in the study area. Prevalence of infection in school age children was used as a proxy to estimate baseline community prevalence and subsequent treatment coverage [25] . The initial parasitological survey was followed by a post-treatment survey in May 2008, six months after the baseline survey and drug distribution. Through the school leadership, parents of pupils in class 4 were requested to come to the school compound to be informed about the study and to provide consent for their children’s participation. Only children of parents who agreed to come to school and gave informed consent and children who gave assent were included in the study. Up to 99% of parents signed the consent forms. A total of 492 children were included in the study following consent by parents and assent by the children. To assess parasite infections at each survey time, children were asked to provide a single stool sample. Two slides were prepared from each stool sample using the Kato-Katz method and each slide was read by an experienced microscopist. Blood was collected by finger-stick to evaluate hemoglobin (Hb) levels using a Hemacue (Ängelholm, Sweden). Socioeconomic data were also collected from parents of participating children using standardized questionnaires in order to determine if prevalence and CDI uptake levels were related to socio-economic status.

### Community drug distributors (CDD)

The CDDs were lay persons from the study area who volunteered for this role. CDDs were trained to administer informed consent during census and drug distribution exercises, to distribute the drugs, and to report back to the health facility and researchers. A training curriculum modeled on the Kenya Ministry of Health training kit for teachers was developed and used for the training of CDDs. Each CDD received a manual that included basic information about the biology of schistosomes and STHs, treatment guidelines, the goals of CDI and the role of a CDD. CDDs were trained for conducting a census by the research team and local Ministry of Health workers. The CDDs obtained consent from compound heads for the census and of all community members for treatments in the door-to-door drug distribution. The research team administered all other consent, including that for the baseline survey among parents and parasitological survey in children.

### Drug distribution

Praziquantel and albendazole were used for the integrated treatment of schistosomes and STH infections, respectively, among residents aged ≥ 5 years, using trained community drug distributors (CDD).

Drug distribution treatment was recorded in the same booklet used for the census, allowing for easy follow-up by the CDDs. Doses of drugs were distributed door-to-door to individuals based on their height, excluding children under 5 years old. The drug distribution exercise carried out in December 2007-February 2008 was disrupted by the post-election skirmishes of late December 2007 –January 2008, but some CDDs who had not completed the exercise were able to resume in February 2008.

### Praziquantel dosage determination by height

We modified the tablet pole [26,27] to a version that was light and foldable and therefore very easy to carry by CDDs. The tablet pole had a magnet at the hinge which held the pole firmly straight when unfolded during use. The pole was color coded for easy determination of drug dosage by the CDDs. CDDs recorded the number of drug tablets given to each individual against height by color code on the pole.

### Data analysis

Quantitative data were entered and summarized using MS Access and Excel. Data were analyzed in MS Access, GraphPad Prism and Excel. Pre-treatment and post-treatment prevalence and infection intensity levels were compared using Chi-square and nonparametric t-tests. P values < 0.05 were considered significant.

### Ethical considerations

This study was reviewed and cleared by the Kenya National Ethics Committee and permission was obtained from District (Bondo) and Divisional (Uyoma) Administration as well as District Education Officers. The project was then introduced to schools through school heads and to the community during community meetings normally called by the location chiefs and village heads. These traditional “*barazas”* (Chief’s meeting) were used for project introduction and information dissemination so as to keep within the normal community structures to ensure sustainability. No parallel community gatherings were introduced. During these meetings, health education was provided to the community. Community members identified one or two CDDs per village, depending on the population.

## Results

### Community census

Results from the census exercise completed by CDDs in October 2008 indicated that there were 5987 adults (persons over 18 years; 57.9% of the population), 3677 (35.5%) children over 5 years and 683 (6.6%) were children under five in East Uyoma, bringing the total population to 10347 and population eligible for treatment to 9664. East Uyoma Location had 25 villages, some of which were sub-divided for our drug distribution convenience, and about 2046 home compounds. According to the 2009 national census data from Kenya Bureau of Statistics, the community had a total population of 15281 which included 7562 (49.5%) males and 7719 (50.5%) females, with 9.6% (n = 1467) of the population being children under 5 and the population eligible for mass treatment being 13814. These data suggest that the CDD census missed about 4934 persons, representing 32.3% of the population.

### Socio-economic survey

Areas endemic for schistosomiasis and STH infections are usually associated with inadequate sanitation and generally lower living standards. To establish the socioeconomic and water contact levels in relationship to CDI uptake, a socio-economic questionnaire was administered to parents of children in class 4 of all 10 primary schools in the East Uyoma location. Of 404 respondents, the majority of community members (95.3%) lived on their own land and practiced subsistence farming. Almost everyone (98.3%) in the community had daily contact with Lake Victoria (34.2%), a pond/dam (45.4%) or creek/river (18.6%), all potential schistosomiasis transmission sites. Only 36.2% of respondents had access to piped water or a well, whether in their homes (9.9%) or in public/community-owned water sources (26.3%). Sanitation was generally poor. While 60.3% of people surveyed had pit toilets, 30.2% did not have access to any sanitation facility and used the open ground for defecation. (Table 1). Less than 2% of the study population owned either a car, refrigerator or had electricity, but a large proportion owned radios (85.1), mobile (cell) phones (35.2%) or bicycles (70.5%). Only 9.7% of people who answered the survey had television sets in their homes. The majority of the houses had dirt/earth flooring (82.4%). Natural/grass roofing was utilized on 42.2% of the houses and 56.1% had corrugated iron roofing. Previous interventions for parasitic diseases in the area had resulted in limited success with 41.7% of survey participants having bed nets in their homes, 68.4% of those nets had been treated regularly. No infections were found among the few children from families that had electricity or owned a car or a refrigerator. However, we did not find any relationship between overall socio-economic status Z scores and infection levels in individual children, prevalence level by village, or CDI coverage. This is perhaps because all the villages were close to the lake (all within 5KM of the shore) and exposure levels among children were not significantly different.

**Table 1:** Relationship between socio-economic characteristics and prevalence of schistosomiasis and geohelminthic infections in school children

**Socio-economic data n = 404**			**Frequency and distribution of parasites% (n)**	
	**N**	**%**	***S. mansoni*** **% (n)**	**Any STH (HW, TT, Asc)% (n)**
**Water Contact: Lake Victoria**				
Everyday	138	34.2	18.25 (23)	14.96 (19)
Sometimes	82	20.3	25.00 (17)	20.59 (14)
Rarely	64	15.9	12.96 (7)	25.93 (14)
Never	119	29.5	12.62 (13)	21.36 (22)
**Pond or Dam**				
Everyday	183	45.4	10.49 (17)	17.79 (29)
Sometimes	59	14.6	20.75 (11)	20.75 (11)
Rarely	35	8.7	17.24 (5)	34.48 (10)
Never	120	29.8	24.00 (24)	17.00 (17)
**Creek or River**				
Everyday	75	18.6	17.39 (12)	15.94 (11)
Sometimes	55	13.6	22.73 (10)	25.00 (11)
Rarely	43	10.7	14.63 (6)	21.95 (9)
Never	220	54.3	15.14 (28)	19.46 (36)
**Drinking water**				
Own pipe	23	5.7	8.70 (2)	17.39 (4)
Public pipe	106	26.3	11.32 (12)	20.75 (22)
Own Well	17	4.2	23.53 (4)	5.88 (1)
Public well	52	12.9	5.77 (3)	15.38 (8)
Rain water	65	16.1	10.77 (7)	6.15 (4)
River/canal	85	21.1	12.94 (11)	12.94 (11)
Other	121	30.0	18.18 (22)	11.57 (14)
**Type of toilet**				
Own flush	2	0.5	50.00 (1)	0
Shared flush	1	0.3	0	0
Pit latrine	244	60.3	12.30 (30)	17.62 (43)
VIP	1	0.3		
Bush/field	146	36.2	18.49 (27)	19.05 (24)
other	5	1.2	0	0
**Housing**				
Electricity	7	1.7	0	0
Fridge	1	0.3	0	0
Car	1	0.3	0	0
Telephone	142	35.2	17.65 (21)	21.74 (25)
Radio	344	85.1	17.06 (50)	19.73 (58)
Bicycle	285	70.5	18.57 (44)	18.91 (45)
TV	39	9.7	11.54 (3)	23.08 (6)
Motor bike	3	0.7	0	19.54 (68)
Domestic worker	27	6.7	9.09 (2)	22.73 (22)
SES quintile (1–4)	142	42.13	16.20 (23)	22.54 (32)
SES quintile (5)	195	57.86	15.38 (30)	18.46 (36)

Socio-economic data was obtained by administering questionnaire to parents. HW – Hookworm, T.T – *T. trichura*, Asc. – *A. lumbricoides,* SES –socioeconomic status.

### Disease distribution

**Figure 2:**
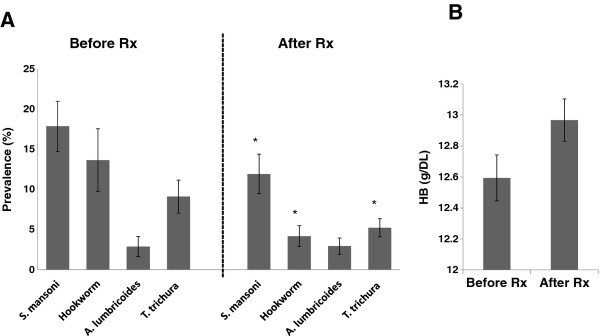
**Comparison of parasite prevalence levels following one round of mass drug distribution.A**. Prevalence levels for *S. mansoni*, hookworm, *A.lumbricoides* and *Trichuris trichura* compared before and after treatment with praziquantel and albendazole. Error bars represent standard deviation (SD) of means. * represents significant change in prevalence following treatment **B**. Haemoglobin levels compared before and after treatment.

Pre-treatment prevalence levels of *S. mansoni* in East Uyoma schools ranged from 5% to 43.2% with an average prevalence of 17.8%. Mean intensity (± SD) of eggs per gram (EPG) was 166.8 ± 295.1 (range 12–1560; CI: 99.8 - 222.0). At the six month follow-up, 576 children were surveyed. *S. mansoni* infections were detected in 38 out of 84 new children who had recently joined the schools and had not participated in the baseline survey. This migration was mostly caused by the post-election violence and led to higher post-treatment prevalence levels in two schools. When the comparison was limited to children surveyed at baseline, there was a 33.2% reduction in mean school prevalence of *S. mansoni* (P =0.04), a 69.4% reduction in hookworm prevalence (P = 0.003), a 1.8% decrease in prevalence of *A. lumbricoides* (NS) and a 42.6% reduction in *T. trichiura* prevalence (P = 0.027) (Figure 2A). Mean intensity (± SD) of *S. mansoni* infection EPG after mass treatment was reduced to 60.3 ± 70.9 (range 12–322; CI: 7.6-46.9). Mean Hb levels were 12.5 ± 1.9 g/dl and ranged from 5.1 g/dl to 18.5 g/dl. Hemoglobin levels increased by 0.4 g/dl from baseline, but the difference was not statistically significant (Figure 2B).

### Household survey

The household survey was used to assess treatment coverage and occurrence of side effects. Sixteen villages were randomly selected for a household survey and assigned to 10 interviewers who walked door-to-door administering post-treatment questionnaires to households. The interviewers were CDDs from different villages from those they were assigned to survey. Each interviewer was asked to randomly interview 50 compounds in each selected village, but an average of only 35.7 (range 13–50) compounds were visited per village, totalling 785 compounds. At the end of 2 weeks, records were retrieved from the interviewers and analyzed. Reasons for the low coverage of compounds by some of the CDDs included interviewer’s inability to access some homes and the slow pace and poor performance of some of the community interviewers. According to the CDD house-hold survey records, the average age of the head of compound was 53.8 years (31.1 -59.6 years). The 785 compounds included in the household survey had an average (± SD) 4.0 households and 12.6 ± 2.6 persons per compound. An average of 7.9 ± 2.0 persons per compound reported receiving treatment, 5.9 ± 3.1 persons reported not receiving treatment and an average of 1.2 ± 0.4 persons per compound were reported absent.

### Treatment coverage

Treatment coverage for praziquantel and albendazole was assessed from CDD registers, but since coverage values obtained from the community registers of the CDDs may be unreliable[28], coverage was also assessed through a household survey using a questionnaire for parents/household heads. The questionnaires were administered in randomly selected households in the communities and administered to all members of the household present at the time of the survey. A total of 8103 persons were treated according to CDD registers. Treatment coverage was calculated as the total number of persons treated divided by total eligible population (persons over 5 years of age) and expressed as a percentage. The percentage of persons treated according to CDD records ranged from 54.2% - 96.6% in individual villages (77.5% ± 16.8; n = 8103). In six villages (Matera, Ukunja, Migowa, Okela, Osewere, and Kakremba), incomplete CDD records were returned after one CDD found a different job before completing the treatment exercise (Matera village); 4 CDDs were offered paid jobs as election clerks in the area before they could complete the treatment exercise and one CDD migrated away from the area (Kakremba village). Other volunteers completed the exercise in those areas after our surveys. Coverage in other areas where treatment was completed ranged from 52.1% - 90.3%. There was a significant difference between coverage by CDD records, 77.5 ± 16.8%, and coverage per total population determined by the household survey, 58.2% (P < 0.01; ANOVA)), which was also comparable to the coverage when official national census population data were used as the denominator (53.0% of the total population and 58.6% of the eligible population). Because the area was mapped before the treatment exercise and all the home compounds in the location were recorded, it was possible to estimate coverage from the CDD records by analyzing the number of compounds visited. This revealed that even though a CDD record may show very high coverage, such coverage was only based on those compounds that had been visited and recorded by the CDD and not necessarily all compounds. According to this analysis, Nyamboi and Kayundi had compound coverage of 23.4% and 33.1% but treatment coverage of 77.5% and 75.9% respectively (Table 2). 

**Table 2:** Drug distribution coverage by homestead compounds visited, CDD records, and household survey (HHS)

**Village**	**Number of Compounds**	**Percent (%) compounds visited**	**n (persons)**	^α^**Percent% coverage by CDD Record**	^α^**Percent% coverage by HHS**
Agege	35	67.7	710	71.1	75.9
Andom A	26	87.7	372	61.3	52.1
Andom B	22	90.5	227	54.2	57.4
Chianda	26	52.3	231	80.1	74.8
Got-Kachola	21	85.6	616	89.6	90.3
^**β**^**Kakremba**	**50**	**74.6**	**438**	**95.7**	**20.1**
Kasiri	16	71.9	679	80.3	ND
Kayundi	49	38.1	162	75.9	ND
Kunya	28	67.6	558	77.4	61.4
Magombe	25	77.5	612	91.1	89.0
Maranba	51	61.6	397	54.9	62.2
^**β**^**Matera**	**50**	**67.2**	**449**	**26.7**	**ND**
^**β**^**Migowa**	**30**	**59.7**	**451**	**86.9**	**37.3**
Mituri	21	80.4	503	73.6	71.3
Mochi	98	77.8	394	98.5	ND
Nyagunda	26	64.2	552	73.2	62.4
Nyamasore A	21	50.7	196	61.2	ND
Nyamasore B	30	64.6	173	77.5	ND
Nyamboi	48	23.4	71	77.5	ND
Ochienga	50	90.0	446	68.6	ND
Odede	25	85.2	388	93.3	55.9
Okela	25	63.3	553	81.2	61.4
^β^**Ukunja**	**50**	**77.5**	**320**	**95.6**	**35.7**
^β^**Osewre**	**35**	**91.9**	**235**	**96.6**	**49.0**
Wayaga	13	81.4	614	94.9	ND
**Total or Average**	**871**	**67.53**	**10347**	**77.47**	**58.1641**

^α^ P = 0.0047 (Paired t) for comparison between CDD record for coverage and independent household survey.

^β^ CDDs quit exercise before completing drug distribution.

### Side effects

Praziquantel is generally safe and usually only causes minor side effects that usually resolve with minimal management. Side effects following treatment were recorded during the household survey. Reported side effects were minor with 12.7% (n = 279), 34.2% (n = 752), 4.7% (n = 103) and 0.3% (n = 21) of those treated reporting diarrhea, fatigue, abdominal pain and vomiting, respectively. Fatigue was the most common reported side effect. Overall, 48.7% of those treated reported one or more of these minor side effects.

### Drug costs

The financial cost of praziquantel for treated persons was $9,865.85, for an average cost per treatment of $0.98, while the cost for albendazole treatment was $0.23 per dose. These drugs were purchased by the project because at the time of the study, free or subsidized drugs were not available from the public health sector. Economic costs associated with the drug distribution were not evaluated as part of this study.

## Discussion

With renewed interest in the control of schistosomiasis and other neglected tropical diseases (NTDs), both by global sponsors and governments in endemic regions, evidence based data on cost effective strategies for mass drug administration are needed to inform control and elimination program managers. Adhering to strategic guidelines issued by the World Health Organization, the main approach has been to use school-based mass treatment for controlling schistosomiasis in highly endemic areas. However, it is recognized that this approach leaves out other members in the community who are also infected and who may be contributing to continued transmission. In recent years, community based treatment has been tested as an alternate approach but community-level programs may be limited by inadequate health care infrastructure at the primary level in many areas of Africa [29,30]. In Kenya, although the government recognizes the importance of local interventions and having policies for preventive approaches for health care, allocation of health resources has been skewed in favor of tertiary and secondary facilities that offer curative services. This approach ignores rural health facilities that are the first point of patient contact and provide the bulk of the healthcare services [31].

As an example of a primary health care level program, CDI has been evaluated and found to be highly successful in reaching persons at risk of disease within the community [32]. CDI emphasizes the significant role of communities as lead partners and is the fundamental factor that has been responsible for the success of APOC. Systematic reviews have shown that the use of community or lay health workers who are trained in the context of the intervention but who have no formal professional or tertiary education in preventative health care, showed promising benefits. Compared with usual care, lay health workers increased the uptake of childhood immunization, promoted breastfeeding, reduced childhood mortality and morbidity from common childhood illnesses, and improved outcomes of tuberculosis treatment [33]. In a study by Ndyomugyenyi and Kabatereine [18], integrated CDI achieved higher treatment coverage (85%) for praziquantel and mebendazole compared to a school-based treatment approach (79%) for children aged 5–14 years. Moreover, CDI is able to reach other at risk groups in the community outside of the school system. However, more controlled studies comparing the CDI and school-based mass-drug administrations in endemic areas are needed.

The aim of this project was to pilot and evaluate the CDI approach for treatment against schistosomiasis and STHs in a rural setting in Nyanza Province, western Kenya, which is outside the APOC zone and therefore untested for CDI. In the current study, the CDDs visited 67.5% of the mapped compounds and achieved an overall coverage of 58.2% as determined by household survey. The effectiveness of CDI coverage was influenced by several factors. Coverage depended on accurate mapping of the targeted treatment area by compound because CDD records were based on only the compounds that the CDD had visited; as a result, they overestimated coverage (Table 2). Further, sociopolitical factors may have influenced the study results and the success of CDI. This CDI coincided with the 5-yearly election period in Kenya, which presented a difficult challenge to many CDDs. The political tension in the community affected the general acceptability of CDDs in homes where they were perceived to be of different political persuasions. Moreover, the community focus on election campaigns led to increased absenteeism from homes. Even with these difficulties, many villages were able to achieve appreciable coverage levels.

In this study, the praziquantel cost was $0.98 per delivered treatment and the albendazole cost was $0.23 per delivered treatment. These drugs were not provided by the donor-subsidized public health sector where it would have been expected that drug costs would be much cheaper or even free. Reducing the cost of praziquantel should be an encouragement to both private and public sectors to implement mass drug distribution programs. These results confirm that praziquantel is well tolerated, causing only minor side effects. However, community wide rumors on drug reactions can adversely affect well organized mass treatments. To help alleviate fears of drug reactions in communities and promote MDA compliance, large-scale control programmes should include pharmacovigilance studies as part of ongoing monitoring and evaluation strategies. Detailed pharmacovigilance data that are disseminated back to the community will provide accurate information to communities about the exact nature, extent and cause of side effects as well as help to discriminate true side effects from other underlying conditions and exaggerated rumors about drug reactions.

## Conclusion

The impact of this one round of drug distribution in a troubled area confirms the feasibility of using CDI for the control of helminth infections in endemic communities. A recently concluded multi-country study on CDI in areas already participating in onchocerciasis control [16] has demonstrated that the CDI process/approach can be effective and feasible for integrated delivery of different health interventions in rural Africa [16,19]. Future testing of CDI should be aimed at testing how CDI may be used widely for multiple interventions to strengthen primary health care at the national level and could integrate with other established control programs such as HIV, TB, malaria and maternal and child health (MCH) programs. As the Kenya Ministry of Health continues to roll out the Community Health Strategy, the future role of CDDs will be taken up by community health workers and be more integrated with other community level health activities. It will be helpful to evaluate these developments as they occur. Currently, we are conducting studies to test how elements of the CDI may be used to strengthen the Kenya Ministry of Health’s community strategy and thereby improve primary health care in rural Kenya. Similar studies in the same setting are comparing community wide treatment to school based treatment in a randomized controlled trial. We are also preparing reports on qualitative findings describing community acceptability of the CDI approach in this setting. This pilot study paves the way for larger scale trials to compare community wide treatments to school-based strategies in the country.

## Competing interests

The authors have no interests to declare.

## Authors’ contributions

PNMM provided overall study supervision, data analysis and prepared the manuscript. SPM carried out sample size calculations and data analysis. COO and EMM were involved with community mobilization and data collection. PNMM, MM, JGA, KFL, EMM, WES, DMSK designed the study. All authors reviewed analyzed data and approved the manuscript.
